# Atom Search Optimization with Deep Learning Enabled Arabic Sign Language Recognition for Speaking and Hearing Disability Persons

**DOI:** 10.3390/healthcare10091606

**Published:** 2022-08-24

**Authors:** Radwa Marzouk, Fadwa Alrowais, Fahd N. Al-Wesabi, Anwer Mustafa Hilal

**Affiliations:** 1 Department of Information Systems, College of Computer and Information Sciences, Princess Nourah bint Abdulrahman University, P.O. Box 84428, Riyadh 11671, Saudi Arabia; 2 Department of Mathematics, Faculty of Science, Cairo University, Giza 12613, Egypt; 3 Department of Computer Sciences, College of Computer and Information Sciences, Princess Nourah bint Abdulrahman University, P.O. Box 84428, Riyadh 11671, Saudi Arabia; 4 Department of Computer Science, College of Science and Art at Mahayil, King Khalid University, Muhayel Aseer 63311, Saudi Arabia; 5 Department of Computer and Self Development, Preparatory Year Deanship, Prince Sattam bin Abdulaziz University, AlKharj 16242, Saudi Arabia

**Keywords:** quality of life, disabled persons, sign language recognition, deep learning, atom search optimization

## Abstract

Sign language has played a crucial role in the lives of impaired people having hearing and speaking disabilities. They can send messages via hand gesture movement. Arabic Sign Language (ASL) recognition is a very difficult task because of its high complexity and the increasing intraclass similarity. Sign language may be utilized for the communication of sentences, letters, or words using diverse signs of the hands. Such communication helps to bridge the communication gap between people with hearing impairment and other people and also makes it easy for people with hearing impairment to express their opinions. Recently, a large number of studies have been ongoing in developing a system that is capable of classifying signs of dissimilar sign languages into the given class. Therefore, this study designs an atom search optimization with a deep convolutional autoencoder-enabled sign language recognition (ASODCAE-SLR) model for speaking and hearing disabled persons. The presented ASODCAE-SLR technique mainly aims to assist the communication of speaking and hearing disabled persons via the SLR process. To accomplish this, the ASODCAE-SLR technique initially pre-processes the input frames by a weighted average filtering approach. In addition, the ASODCAE-SLR technique employs a capsule network (CapsNet) feature extractor to produce a collection of feature vectors. For the recognition of sign language, the DCAE model is exploited in the study. At the final stage, the ASO algorithm is utilized as a hyperparameter optimizer which in turn increases the efficacy of the DCAE model. The experimental validation of the ASODCAE-SLR model is tested using the Arabic Sign Language dataset. The simulation analysis exhibit the enhanced performance of the ASODCAE-SLR model compared to existing models.

## 1. Introduction

Communication is the major component of interpersonal relationships that acts as an important connection between individuals and describes human existence. Additionally, it is a prominent basis to promote the growth of the human population. Communication is classified into verbal and nonverbal forms, and its core is to exchange data between the sender and the receiver [1]. As the component of communication, verbal and non-verbal forms are considered spontaneous and disguised spontaneous communications, the initial one is demonstrated as an intentional communication from the motivation emotional state, and the last one is demonstrated as an instinctive intentional strategic operation [2]. Communication is an indispensable tool in the existence of human beings. It is an effective and fundamental method of sharing opinions, thoughts, and feelings. However, a considerable fraction of the world population lacks this capability [3]. A lot of people suffer from speaking impairment, hearing loss, or both. A complete or partial disability to hearing in one or both ears is called hearing loss. In contrast, being mute is an inability that impairs speaking and makes people have difficulty speaking [4]. During childhood, if deaf–mute happens, their language learning capability could be hindered and leads to language impairment, otherwise called hearing mutism.

Sign language (SL) is the major adaptation for people with hearing and speech disabilities. Additionally, it is called a visual language. In general, it contains five key characteristics: orientation, hand shape, location, movement, and components such as eyebrow movements and mouth shape [5]. Studies had been carried out on voice generation using smart gloves that may provide a voice to SL movement. However, those who do not know SL generally reject or undervalue persons with disability due to the lack of proper communication among themselves. The procedure of translating the gestures and signs portrayed by the user into text is called SL detection [6]. It links the communication gap between the general public and people who could not speak. Image processing algorithms and neural networks are used for mapping the gesture to proper text in the training dataset and thus raw videos or images are transformed into relevant text that cannot be understood and read [7].

Existing models have used statistical approaches and machine learning (ML) models for SL recognition. The ML models are based on handcrafted features which could not determine insignificant regions in every frame, and the existence of temporal misalignment makes it difficult for traditional approaches to determine robust features. The derived features encode temporal dependency among frames, position, and orientation of hands, face, etc. Background noise and varying lighting conditions also result in occlusions and clutter, which have to be considered. They are tedious to extract with traditional ML models. Therefore, in this study, we have proposed a deep learning (DL)-based model as a solution for SL recognition. The primary objective of the DL technique is automated feature engineering. The concept behind this is to learn a set of features automatically from raw information that is beneficial during SL detection [8]. In such a way, it prevents the manual method of hand-crafted features by automatically learning as a set of features. With the emergence of the DL method, end to end model has been constructed for numerous challenges that only need the image as input [9]. Lately, a large number of studies have been ongoing in developing a system that is capable of classifying signs of various SLs in the class. This system has found application in natural language communications, games virtual reality environments, and robot controls [10].

This study designs an atom search optimization with a deep convolutional autoencoder-enabled Arabic Sign Language recognition (ASODCAE-SLR) model for speaking and hearing disabled persons. The presented ASODCAE-SLR technique preprocesses the input frames by a weighted average filtering approach. In addition, the ASODCAE-SLR technique employs a capsule network (CapsNet) feature extractor. For the recognition of sign language, the DCAE model is exploited in the study. At the final stage, the ASO algorithm is utilized as a hyperparameter optimizer which in turn increases the efficacy of the DCAE model. The experimental validation of the ASODCAE-SLR model is tested using the Arabic Sign Language dataset.

## 2. Literature Review

Sruthi and Lijiya [11] proposed a signer-independent DL-based method to build a sign language (SL) static alphabet detection technique. Now, the study examines many prevailing models in SL detection and carries out a CNN structure for ISL static alphabet detection from the binary silhouette of the signer hand area. Wen et al. [12] developed an AI-assisted SL detection and transmission method encompassing a virtual reality interface, sensing gloves, and DL block. Segmentation and non-segmentation enabled DL algorithm to accomplish the detection of 20 sentences and 50 words. The segmentation technique classifies whole sentence signals into word units. Next, the DL method identifies each word element and reversely recognizes and reconstructs sentences. Khan et al. [13] aimed to illustrate a user-friendly method for Bangla SL for converting text via CNN and personalized ROI segmentation. By utilizing the ROI selection approach, the technique illustrates improved performance when compared to traditional methodologies.

In [14], the authors developed an SL fingerspelling alphabet detection technique with an image processing technique, supervised deep learning, and machine learning. Especially, twenty-four alphabetical symbols are developed by different integrations of static gestures (not including two motion gestures Z and J). Local binary pattern (LBP) and histogram of oriented gradients (HOG) features of every gesture would be extracted from the training image. Next, the multi-class support vector machine (SVM) is employed for training the extracted dataset. Mannan et al. [15] applied a deep convolution neural network for ASL alphabet detection to resolve ASL detection problems. The study proposed an ASL detection technique with a DCNN. The efficiency of the DCNN method enhances the quantity of the given dataset; for these purposes, we employed the data augmentation method for expanding the size of the trained dataset from the current dataset. 

Sharma et al. [16] introduce a DCNN method for recognizing different symbols in ISL, which belongs to thirty-five classes. Such classes comprise cropped images of hand gestures. Different from other feature selection-based models, DCNN has the benefit of automated feature extraction in the training. It is named end-to-end learning. A lightweight transfer learning (TL) structure makes the model training faster which provides 100% accuracy. Furthermore, a web-based method was proposed which could simply decode the symbol. In [17], a proposed novel architecture for signer-independent SL detection with different DL architectures encompassing DRNN, hand semantic segmentation, and hand shape feature representation. Extracting hand shape features can be accomplished by a single-layer convolution self-organizing map (CSOM) rather than depending on the TL of pretrained DCNN. Then, the series of extracted feature vectors are identified by utilizing deep BiLSTM-RNN.

Though several ML and DL models for sign language recognition are available in the literature, it is still needed to enhance classification performance. Owing to the continual deepening of the model, the number of parameters of DL models also increases quickly which results in model overfitting. Since the trial and error method for hyperparameter tuning is a tedious and erroneous process, metaheuristic algorithms can be applied. Therefore, in this work, we employ the ASO algorithm for the parameter selection of the DCAE model.

## 3. The Proposed Model

In this study, a new ASODCAE-SLR technique has been developed for recognizing sign languages to assist the communication of speaking and hearing disabled persons. The ASODCAE-SLR technique initially pre-processes the input frames by a weighted average filtering approach. Next, the ASODCAE-SLR technique employed a CapsNet feature extractor to produce a collection of feature vectors. To identify and classify sign language, ASO with the DCAE model is exploited in the study.

### 3.1. Image Pre-Processing

The ASODCAE-SLR technique initially pre-processes the input frames by a weighted average filtering approach. The weighted average filter was planned to pre-process that suppresses noise and improves spatial domain features efficiently [9]. This filter $W^{\eta }$ was determined as a matrix, whereas η refers to the odd number. All the element values of the matrix were defined as the distance between the present place and the center of the matrix, as demonstrated in Equation (1). The center of the matrix was determined as $w_{\left(\eta +1\right)/2,\left(\eta +1\right)/2}=2/\eta^{2}$. The presented filter continues edges but suppresses speckle noise related to another filter namely the mean filter and maintains the continuity of images.

(1) $$w_{i}j=\frac{1}{\eta^{2}\sqrt{{\left(\frac{\eta +1}{2}-i\right)}^{2}+{\left(\frac{\eta +1}{2}-j\right)}^{2}}};i=1,2,\;\eta ;j=1,2\;\eta ;\;$$

whereas $I_{1},I_{2}\in R^{N_{r}\times N_{C}}$, the convolutional of all the images utilizing $W^{\eta }$ is obtained for acquiring 2 images $I_{1}^{w}\left(\eta \right)=I_{1}*W^{\eta }$ and $I_{2}^{w}\left(\eta \right)=I_{2}*W^{\eta }$, whereas * signifies the 2D convolutional function.

### 3.2. Feature Extraction: CapsNet Model

**Figure 1:**
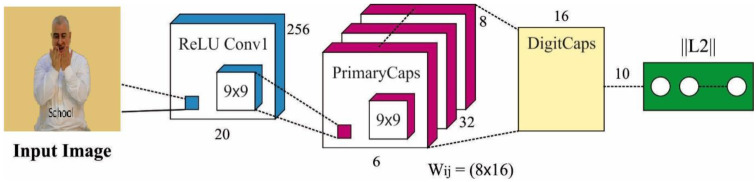
Structure of CapsNet model.

Next to pre-processing, the ASODCAE-SLR technique employs the CapsNet model to generate feature vectors. A major benefit of CapsNet is that they hold the characteristics of more concrete features which can be interpreted to understand what and how is the network learning. The CapsNet has the ability for encoding spatial data and differentiate among several poses, textures, and orientations [18]. The capsule is a set of neurons, thus all the capsules have an activity vector connected to it that captures several instantiation parameters to recognition of a certain kind of object or its part. The length and orientation of the vector present the probability or possibility of the presence of that object and its generalization pose. These vectors were passed on to the upper-level capsules in lower-layer capsules. The coupling coefficients occur among these layers of capsules. When the forecast by the lower-level capsule equals the outcome of current capsules, the value of coupling coefficient amongst them improves, calculated with utilize of softmax function. Specifically, when the present capsule identifies a tight cluster of preceding prediction, strongly representing the occurrence of that object, its outcomes in a higher probability is also recognized as routing by agreement. Figure 1 depicts the framework of the CapsNet method. 

Initially, the prediction vector (Equation (2)) was calculated as:

(2) $${\hat{u}}_{j_{|i}}\wedge =W_{ij}u_{i},$$

whereas ${\hat{u}}_{j_{|i}}$ refers to the outcome of the forecast vector of upper-level jth capsule, $W_{ij}$ and $u_{i}$ implies the weighted matrix and forecast vector of capsules i from the lower layer correspondingly. It can capture spatial connections and interactions among sub-objects and objects. In Equation (3), dependent upon the degree of agreement amongst neighboring layer capsules, the coupling coefficients were calculated using the softmax function,

(3) $$c_{ij}=exp\left(b_{ij}\right)/\overset{\;}{\underset{\;}{\sum }}\;exp\left(b_{ik}\right),\;\;$$

In which $b_{ij}$ signifies the  log probability amongst two capsules, initialization to zero, and k represents the number of capsules. The input vector $s_{j}$ to jth layer capsule that a weighted sum of ${\hat{u}}_{j_{|i}}\wedge$ vectors learned by routing technique is computed as:

(4) $$s_{j}=\overset{\;}{\underset{i}{\sum }}c_{ij}{\hat{u}}_{j_{|i}\;\;},\;$$

Lastly, a squashing function that integrates squashing and unit scaling (Equation (5)) was executed for confining the value of results from the range amongst zero and one, therefore calculating the probability as,

(5) $$\|s_{j}{\|}^{2}v_{j}=\underline{1+\|s_{j}{\|}^{2}}\frac{s_{j}}{\left\|s_{j}\right\|},$$

The loss function (as calculated by Equation (6)) was connected to capsules from the final layer, whereas m+arιd m- are fixed to 0.9 and 0.1 resp.

(6) $$l_{k}=T_{k}\;max\;{\left(0,\;m^{+}-\left|\left|v_{k}\right|\right|\right)}^{2}+\lambda \left(1-T_{k}\right)\;max\;{\left(0,\;\left|\left|v_{k}\right|\right|-m^{-}\right)}^{2},\;\;\;\;\;\;\;\;\;$$

whereas the value $T_{k}$ is 1 for correct labels and 0 else, λ refers to the constant whose value is 0.5. The 1st term is calculated to correct labels, and the second term calculates to incorrect labels. If $T_{k}$ will be 1, the second term develops 0, and for $T_{k}$ as 0, the first term develops 0. Similarly, the loss value $1_{k}$ is 0 for correct forecasts with $v_{k}$ being superior to 0.9 and non-zero otherwise.

### 3.3. Sign Language Recognition: DCAE Model

To identify and classify sign language, the DCAE model is exploited in the study. AE is a conventional DNN structure that makes use of its input as a label. Later, the network attempts to recreate its input in the learning mechanism [19]; for these purposes, it generates and automatically extracts the representation feature in suitable time iterations. This kind of network is created by stacking deep layers in AE forms consisting of two major parts of decoder and encoder. DCAE is a kind of AE applying a convolution layer to determine the inner data of an image. In CAE, structure weight is shared amongst each location within every feature map, thereby reducing parameter redundancy and preserving the spatial locality. For extracting deep features, consider D, W, and H as the depth, width, and height of the dataset, correspondingly, and n refers to the pixel count. For every member of the X set, the image patches with the size 7×7×D are extracted, where $\chi_{j}$ denotes the central pixel. Consequently, the X set is characterized as an image patch, every patch, $x_{i}^{*}$, is given into the encoder blocks. For an input $x_{i}^{*}$, the hidden layer mapping of kth feature map is shown below:

(7) $$h^{k}=\sigma \left(x_{i}^{*}*W^{k}+b^{k}\right)$$

In Equation (7), b refers to the bias; σ denotes an activation function, and the symbol * corresponds to the 2D convolution layer and it is attained by the following expression:

(8) $$y=\sigma \left(\overset{\;}{\underset{k\in H}{\sum }}h^{k}*{\tilde{W}}^{k}+{\tilde{b}}^{k}\right)\;\;$$

In Equation (8), there exists bias $\tilde{b}$ for every input channel, and h denotes the set of latent feature maps. The $\tilde{W}$ corresponding to the flip operation over both dimensions of weight *W*. y denotes the prediction value. In order to define the parameter vector depicting the complete DCAE architecture, one could minimalize the subsequent cost function signified as follows:

(9) $$E\left(\theta \right)=\frac{1}{n}\overset{n}{\underset{i=1}{\sum }}\;\|x_{i}^{*}-y_{i}{\|}_{2}^{2}\;\;$$

For minimizing this function, we need to evaluate the gradient of cost function concerning the convolutional kernel $\left(W,\tilde{W}\right)$ and bias $\left(b,\;\tilde{b}\right)$ parameter:

(10) $$\frac{\partial E\left(\theta \right)}{\partial W^{k}}=x^{*}*\delta h^{k}+h^{k}*\delta y\;$$

(11) $$\frac{\partial E\left(\theta \right)}{\partial b^{k}}=\delta h^{k}+\delta y\;\;$$

Now, δh and δy denote the deltas of the hidden state and the reconstruction, correspondingly. Then, the weight is upgraded by the optimization methodology. At last, the DCAE parameter is evaluated when the loss function convergence is accomplished. The output feature map of the encoder block is regarded as a deep feature. In the study, batch normalization (BN) was employed for tackling the internal covariant shift phenomenon and enhancing the efficiency of the network via the normalization of input layers by re-centering and rescaling. The BN assists to increase accuracy and learn faster.

### 3.4. Hyperparameter Tuning: ASO Algorithm

In this study, the ASO algorithm is exploited to finely adjust the hyperparameter values related to the DCAE model. The molecular dynamics simulate the mathematical process of the ASO technique. In ASO, the place of all the atoms from the searching space that is affected by their mass signifies the solutions [20]. ASO begins the optimization by creating a group of arbitrary particles from N-dimensional space. Afterward, the solution of all the atoms was estimated as dependent upon the main function. Atoms upgrade their place and velocity from all the iterations, and the place of the optimum atom was upgraded from all the iterations. The velocity of particles is a function of their acceleration, and the acceleration of atoms is estimated based on Newton’s second law dependent upon the ratio of forces executed to the mass of particles. The mass of $i^{th}$ atom from the iteration of t, $m_{i}\left(t\right)$ was computed by the subsequent formulas:

(12) $$M_{i}\left(t\right)=e^{\frac{Fit_{i}\left(t\right)-Fit_{Best}\left(t\right)}{Fit_{Best}\left(t\right)-Fit_{Worst}}}$$

(13) $$m_{i}\left(t\right)=\frac{M_{i}\left(t\right)}{\sum_{j=1}^{N}\;M_{j}\left(t\right)}$$

whereas $Fit_{Best}\left(t\right)$ and $Fit_{Worst}$ signifies atoms with optimum and worse values from the $t^{th}$ iteration and $Fit_{i}\left(t\right)$ implies the value of $i^{th}$ atom main function from the $T^{th}$ iteration, correspondingly. Regarding the minimize problems, $Fit_{Best}$ and $Fit_{Worst}$ were assumed dependent upon the subsequent connections:

(14) $$Fit_{Best}\left(t\right)=min\;\left(Fit_{i}\left(t\right)\right),\;i\in \left\{1,2,\;\ldots ,\;N\right\}\;$$

(15) $$Fit_{Worst}\left(t\right)=max\;\left(Fit_{i}\left(t\right)\right),\;i\in \left\{1,2,\ldots ,N\right\}$$

During all the periods, the count of neighbors of all the atoms that interact is defined utilizing Equation (16):

(16) $$K\left(t\right)=N-\left(N-2\right)\times \left[\frac{T}{T}\;\right]$$

**Figure 2:**
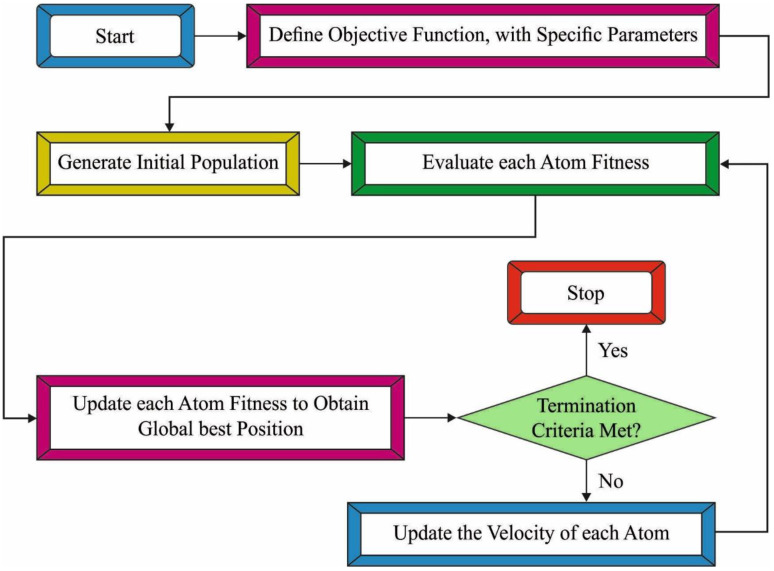
Flowchart of ASO technique.

In which T defines the entire amount of iterations of the technique, or in another word, the life of systems. As is noted, the parameter K is a function of time, slowly reducing the iterations. The forces executed on all the particles contain two kinds of interaction forces and internal constraint forces. The interaction force that is determined utilizing the Lennard–Jones potential method and the internal constraint force that is connected to the bond length potential and differs depending upon the distance amongst all the atoms to optimum atoms were computed utilizing Equations (17) and (18), correspondingly.

(17) $$F_{i}^{d}\left(t\right)=\overset{\;}{\underset{j\in K_{Best}}{\sum }}\;rand_{j}F_{ij}{\left(t\right)}^{d}\;\;\;$$

$$F_{ij}\left(t\right)=-\alpha {\left(1-\frac{t-1}{T}\right)}^{3}e^{-\frac{20t}{T}}[2{\left(h_{ij}\left(t\right)\right)}^{13}-\left(h_{ij}\left(t\right){)}^{7}\right]$$

(18) $$G_{i}^{d}\left(t\right)=-\lambda \left(t\right)\left(x_{best}^{d}\left(t\right)-x_{i}^{d}\left(t\right)\right),\lambda \left(t\right)=\beta e\left(-20\frac{T}{T}\right)$$

whereas F and G define the communication and internal constrain forces correspondingly, $rand_{j}$ depicts an arbitrary number amongst 0 and 1, and $K_{Best}$ refers to the subset of the atom population containing K atoms with optimum main function values. Additionally, $x_{best}^{d}\left(t\right)$ demonstrates the place of an optimum atom from the $t^{th}$ iteration from the d dimensional space, λ(t) illustrates the Lagrangian coefficient, α stands for the depth coefficient, and β implies the weighted coefficient. Figure 2 illustrates the flowchart of the ASO technique.

As follows, the acceleration of i particle from the dimensional d and period τ was computed in Equation (19):

(19) $$a_{i}^{d}\left(t\right)=\frac{F_{i}^{d}\left(t\right)}{m_{i}^{d}\left(t\right)}+\frac{G_{i}^{d}\left(t\right)}{m_{i}^{d}\left(t\right)}=\;-\alpha {(}^{1}\cdot e^{\left(-20\frac{T}{T}\right)}\times \overset{\;}{\underset{j\in Kbest}{\sum }}\;\frac{r_{i}[2\times (\left(h_{ij}\left(t\right){)}^{13}-h_{ij}\left(t\right){)}^{7}\right]}{m_{i}\left(t\right)}\frac{\left(X_{j}^{d}\left(t\right)-X_{i}^{d}\left(\|\right)\right)}{|\left|X_{i}\left(t\right),X_{j}\left(t\right)\right|{|}_{2}}\;+\frac{\beta e^{\left(-20\frac{T}{T}\right)}\left(X_{best}^{d}\left(t\right)-X_{i}^{d}\left(t\right)\right)}{m_{i}\left(t\right)}$$

The last step in all the iterations is for updating the particle velocity and location that is achieved in the subsequent formulas:

(20) $$v_{i}^{d}\left(t+1\right)=rand_{i}^{d}v_{i}^{d}\left(t\right)+a_{i}^{d}\left(t\right)\;$$

(21) $$x_{i}^{d}\left(t+1\right)=x_{i}^{d}\left(t\right)+v_{i}^{d}\left(t+1\right)\;$$

Every update and compute were carried out constantly still the termination condition is met. Lastly, the location and value of the main function of an optimum atom were assumed as the optimum estimate of problems.

The ASO approach derives a fitness function to achieve an enhanced performance of the classification. It defines a positive integer to signify the performance of the candidate solution. The minimization of the classification error rate is considered as the fitness function in this study as follows.

(22) $$itness\left(x_{i}\right)=ClassifierErrorRate\left(x_{i}\right)\;=\frac{number\;of\;misclassified\;samples}{Total\;number\;of\;samples}*100$$

## 4. Result Analysis

The proposed model is simulated using Python 3.6.5 tool on PC i5-8600k, GeForce 1050Ti 4GB, 16GB RAM, 250GB SSD, and 1TB HDD. The parameter settings are given as follows: learning rate: 0.01, dropout: 0.5, batch size: 5, epoch count: 50, and activation: ReLU. This section inspects the sign language recognition outcomes of the ASODCAE-SLR model using the Arabic Sign Language dataset. In this study, a total of 1100 samples under 11 class labels are used. Table 1 depicts the detailed description of the dataset.

**Figure 3:**
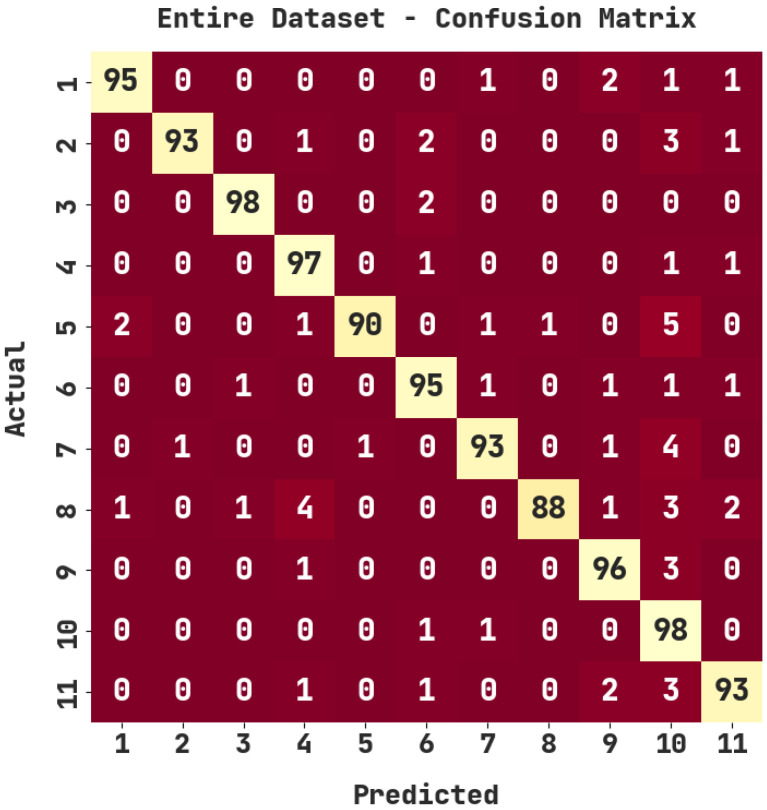
Confusion matrix of ASODCAE-SLR approach under entire dataset.

The confusion matrix generated by the ASODCAE-SLR model on the entire dataset is demonstrated in Figure 3. The figure depicted that the ASODCAE-SLR model has accurately recognized all the 11 class labels on the entire dataset.

Table 2 report the sign language recognition outcomes of the ASODCAE-SLR model on the entire dataset. The ASODCAE-SLR model has recognized samples under class 1 with $accu_{y}$, $prec_{n}$, $reca_{l}$, $F1_{score}$, and Jaccard index  of 99.27%, 96.94%, 95%, 95.96%, and 99.23%. Additionally, the ASODCAE-SLR system has recognized samples under class 2 with $accu_{y}$, $prec_{n}$, $reca_{l}$, $F1_{score}$, and Jaccard index  of 99.27%, 96.94%, 93%, 95.88%, and 92.08%. In line with this, the ASODCAE-SLR method has recognized samples under class 3 with $accu_{y}$, $prec_{n}$, $reca_{l}$, $F1_{score}$, and Jaccard index  of 99.64%, 98%, 98%, 98%, and 96.08%. Next, the ASODCAE-SLR system has recognized samples under class 4 with $accu_{y}$, $prec_{n}$, $reca_{l}$, $F1_{score}$, and Jaccard index  of 99%, 92.38%, 97%, 94.63%, and 89.81%.

**Figure 4:**
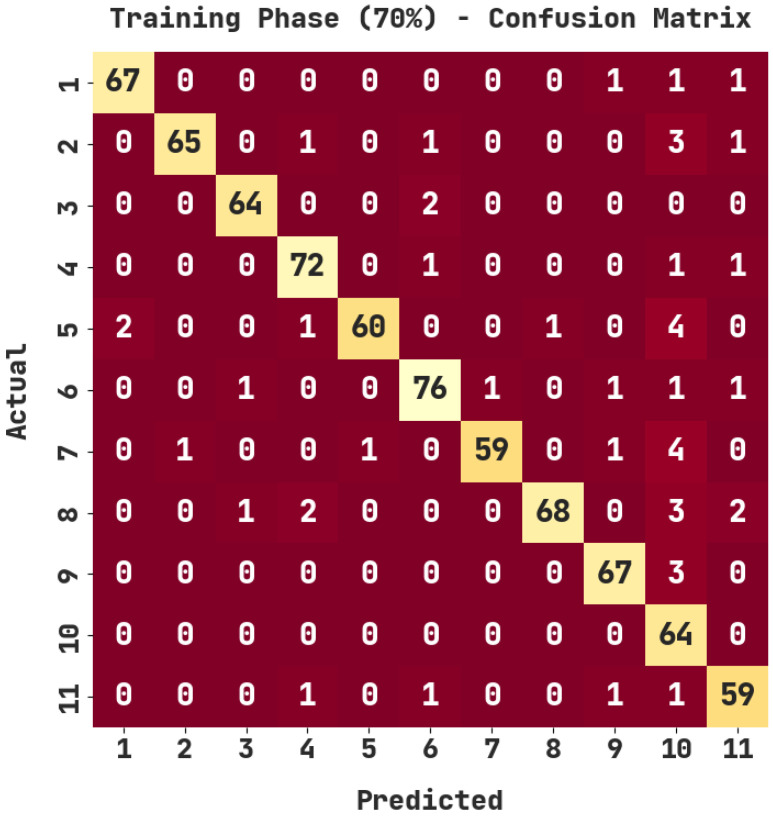
Confusion matrix of ASODCAE-SLR approach under 70% of TR data.

The confusion matrix generated by the ASODCAE-SLR approach on 70% of training (TR) data is displayed in Figure 4. The figure depicted that the ASODCAE-SLR model has accurately recognized all the 11 class labels on 70% of TR data.

Table 3 illustrate the sign language recognition outcomes of the ASODCAE-SLR methodology on 70% of TR data. The ASODCAE-SLR technique has recognized samples under class 1 with $accu_{y}$, $prec_{n}$, $reca_{l}$, $F1_{score}$, and Jaccard index  of 99.35%, 97.10%, 95.71%, 96.40%, and 93.06%. Additionally, the ASODCAE-SLR algorithm has recognized samples under class 2 with $accu_{y}$, $prec_{n}$, $reca_{l}$, $F1_{score}$, and Jaccard index  of 99.09%, 98.48%, 91.55%, 94.89%, and 90.28%. Similarly, the ASODCAE-SLR approach has recognized samples under class 3 with $accu_{y}$, $prec_{n}$, $reca_{l}$, $F1_{score}$, and Jaccard index  of 99.48%, 96.97%, 96.97%, 96.97%, and 94.12%. At last, the ASODCAE-SLR system has recognized samples under class 4 with $accu_{y}$, $prec_{n}$, $reca_{l}$, $F1_{score}$, and Jaccard index  of 98.96%, 93.51%, 96%, 94.74%, and 90%.

**Figure 5:**
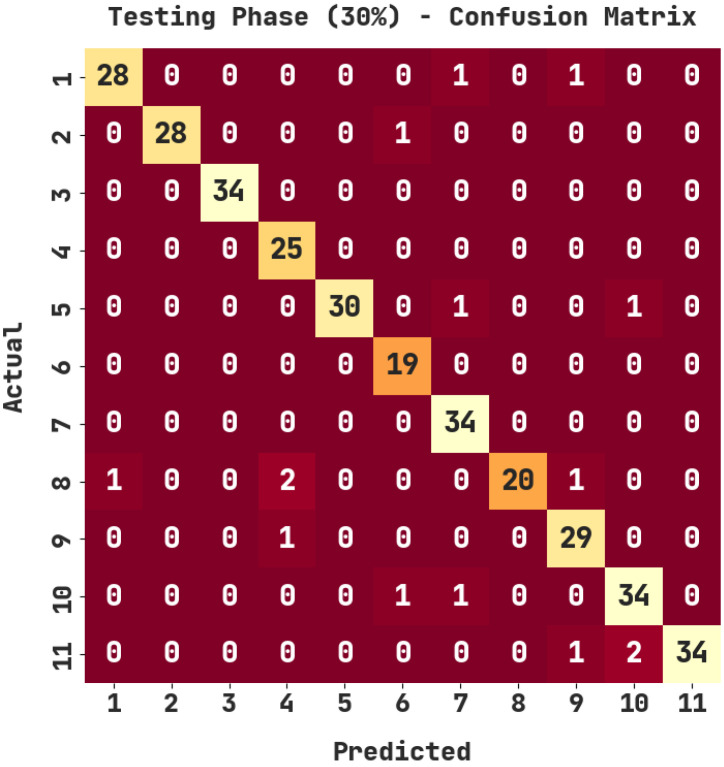
Confusion matrix of ASODCAE-SLR approach under 30% of TS data.

The confusion matrix generated by the ASODCAE-SLR approach on 30% of testing (TS) data is represented in Figure 5. The figure depicted that the ASODCAE-SLR technique has accurately recognized all the 11 class labels on 30% of the TS dataset.

Table 4 demonstrates the sign language recognition outcomes of the ASODCAE-SLR technique on 30% of TS data. The ASODCAE-SLR approach has recognized samples under class 1 with $accu_{y}$, $prec_{n}$, $reca_{l}$, $F1_{score}$, and Jaccard index  of 99.09%, 96.55%, 93.33%, 94.92%, and 90.32%. Furthermore, the ASODCAE-SLR methodology has recognized samples under class 2 with $accu_{y}$, $prec_{n}$, $reca_{l}$, $F1_{score}$, and Jaccard index  of 99.70%, 100%, 96.55%, 98.25%, and 96.55%. In addition, the ASODCAE-SLR system has recognized samples under class 3 with $accu_{y}$, $prec_{n}$, $reca_{l}$, $F1_{score}$, and Jaccard index  of 100%, 100%, 100%, 100%, and 100%. Afterward, the ASODCAE-SLR system recognized samples under class 4 with $accu_{y}$, $prec_{n}$, $reca_{l}$, $F1_{score}$, and Jaccard index  of 99.09%, 89.29%, 100%, 94.34%, and 89.29%.

**Figure 6:**
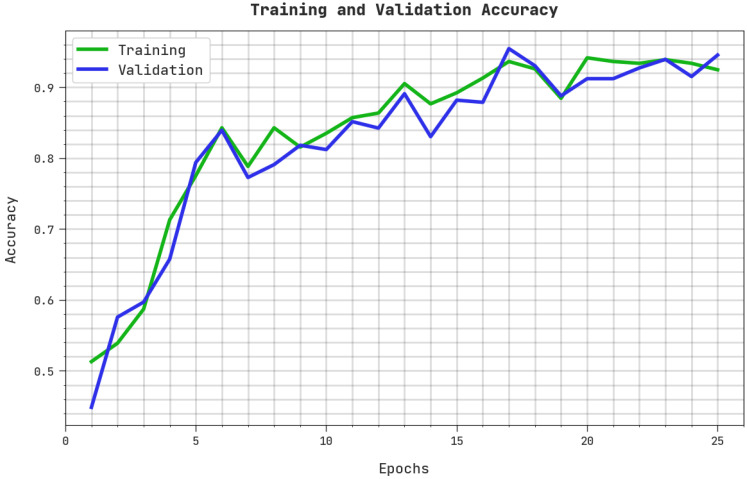
TRA and VLA analysis ASODCAE-SLR approach.

The training accuracy (TRA) and validation accuracy (VLA) acquired by the ASODCAE-SLR approach on the test dataset is shown in Figure 6. The experimental result stated that the ASODCAE-SLR technique has achieved improved values of TRA and VLA. Particularly the VLA seemed greater than TRA.

**Figure 7:**
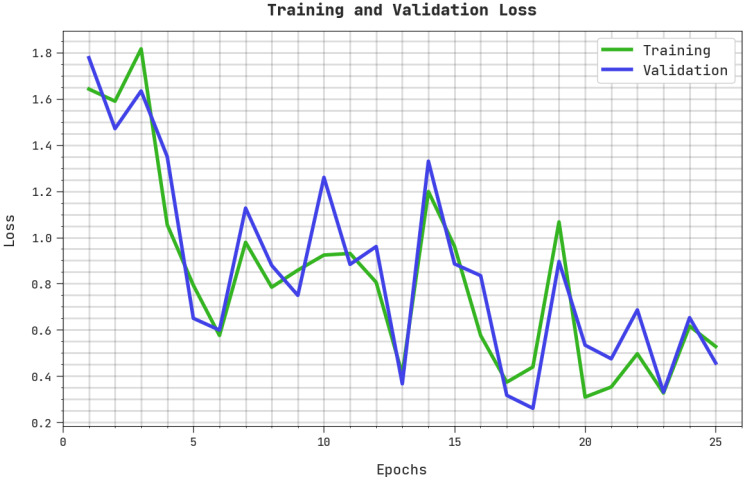
TRL and VLL analysis ASODCAE-SLR approach.

The training loss (TRL) and validation loss (VLL) accomplished by the ASODCAE-SLR system on the test dataset are depicted in Figure 7. The experimental result revealed that the ASODCAE-SLR approach has obtained minimal values of TRL and VLL. Certainly, the VLL is lesser than TRL.

**Figure 8:**
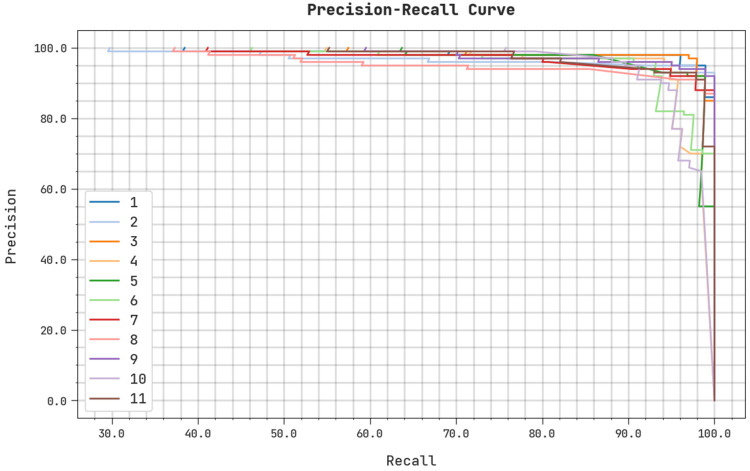
Precision-recall analysis ASODCAE-SLR approach.

A clear precision–recall examination of the ASODCAE-SLR algorithm on the test dataset is illustrated in Figure 8. The figure represented that the ASODCAE-SLR approach has resulted in higher values of precision-–recall values under all classes.

**Figure 9:**
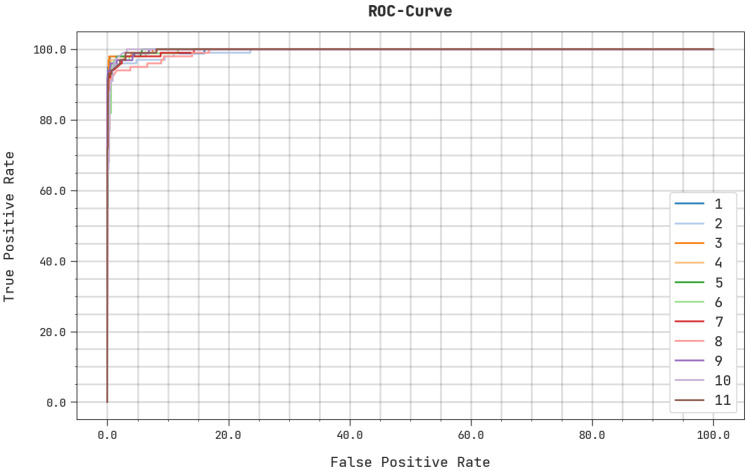
ROC analysis ASODCAE-SLR approach.

A detailed ROC analysis of the ASODCAE-SLR system on the test dataset is illustrated in Figure 9. The outcomes represented by the ASODCAE-SLR algorithm have demonstrated their capability in categorizing distinct classes on the test dataset.

**Figure 10:**
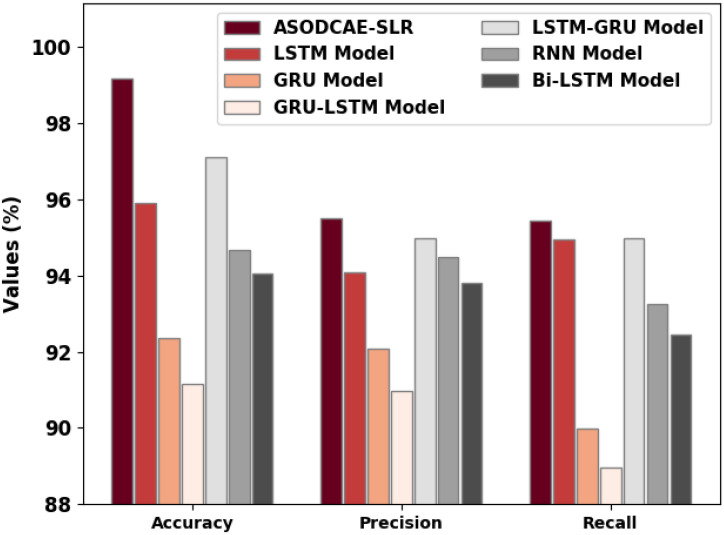
Comparative analysis of ASODCAE-SLR approach with existing algorithms.

At last, comprehensive comparative results of the ASODCAE-SLR model with recent models are given in Figure 10 [21]. The figure indicated that the GRU-LSTM model has attained reduced classification results compared to existing techniques.

Next, the GRU, RNN, and BiLSTM models have reported slightly enhanced classification performance whereas the LSTM model has shown reasonable classification performance. Moreover, the LSTM-GRU model has accomplished near-optimal performance. However, the obtained values implied that the ASODCAE-SLR model has accomplished improved performance over other models.

## 5. Conclusions

In this study, a new ASODCAE-SLR technique has been developed for recognizing sign languages to assist the communication of speaking and hearing disabled persons. The ASODCAE-SLR technique initially pre-processes the input frames by a weighted average filtering approach. Next, the ASODCAE-SLR technique employed a CapsNet feature extractor to produce a collection of feature vectors. To identify and classify sign language, the DCAE model is exploited in the study. At the final stage, the ASO algorithm is utilized as a hyperparameter optimizer which in turn increases the efficacy of the DCAE model. The experimental validation of the ASODCAE-SLR model is tested using the Arabic Sign Language dataset. The simulation analysis exhibit the enhanced performance of the ASODCAE-SLR model compared to existing models. Therefore, the proposed model can be employed to assist communication between deaf and dumb people with ordinary people. The proposed model can be extended to sign board recognition in real-time applications. In the future, the performance of the proposed model can be tested on a real-time large-scale dataset. In addition, a fusion of DL models can be derived to boost the SL recognition performance.

## Figures and Tables

**Table 1:** Dataset details.

Label	Arabic Word	Meaning	No. of Samples
1	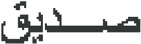	Friend	100
2	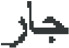	Neighbor	100
3	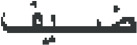	Guest	100
4	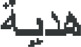	Gift	100
5	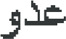	Enemy	100
6	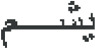	To Smell	100
7	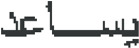	To Help	100
8	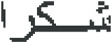	Thank You	100
9	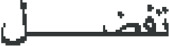	Come in	100
10	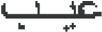	Shame	100
11	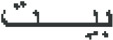	House	100
Total Number of Samples			1100

**Table 2:** Result analysis of ASODCAE-SLR approach with distinct class labels under entire dataset.

Entire Dataset					
Labels	Accuracy	Precision	Recall	F1-Score	Jaccard Index
1	99.27	96.94	95.00	95.96	92.23
2	99.27	98.94	93.00	95.88	92.08
3	99.64	98.00	98.00	98.00	96.08
4	99.00	92.38	97.00	94.63	89.81
5	99.00	98.90	90.00	94.24	89.11
6	98.91	93.14	95.00	94.06	88.79
7	99.00	95.88	93.00	94.42	89.42
8	98.82	98.88	88.00	93.12	87.13
9	99.00	93.20	96.00	94.58	89.72
10	97.64	80.33	98.00	88.29	79.03
11	98.82	93.94	93.00	93.47	87.74
Average	98.94	94.59	94.18	94.24	89.19

**Table 3:** Result analysis of ASODCAE-SLR approach with distinct class labels under 70% of TR data.

Training Phase (70%)					
Labels	Accuracy	Precision	Recall	F1-Score	Jaccard Index
1	99.35	97.10	95.71	96.40	93.06
2	99.09	98.48	91.55	94.89	90.28
3	99.48	96.97	96.97	96.97	94.12
4	98.96	93.51	96.00	94.74	90.00
5	98.83	98.36	88.24	93.02	86.96
6	98.70	93.83	93.83	93.83	88.37
7	98.96	98.33	89.39	93.65	88.06
8	98.83	98.55	89.47	93.79	88.31
9	99.09	94.37	95.71	95.04	90.54
10	97.27	75.29	100.00	85.91	75.29
11	98.70	90.77	93.65	92.19	85.51
Average	98.84	94.14	93.68	93.67	88.23

**Table 4:** Result analysis of ASODCAE-SLR approach with distinct class labels under 30% of TS data.

Testing Phase (30%)					
Labels	Accuracy	Precision	Recall	F1-Score	Jaccard Index
1	99.09	96.55	93.33	94.92	90.32
2	99.70	100.00	96.55	98.25	96.55
3	100.00	100.00	100.00	100.00	100.00
4	99.09	89.29	100.00	94.34	89.29
5	99.39	100.00	93.75	96.77	93.75
6	99.39	90.48	100.00	95.00	90.48
7	99.09	91.89	100.00	95.77	91.89
8	98.79	100.00	83.33	90.91	83.33
9	98.79	90.62	96.67	93.55	87.88
10	98.48	91.89	94.44	93.15	87.18
11	99.09	100.00	91.89	95.77	91.89
Average	99.17	95.52	95.45	95.31	91.14

## Data Availability

Data sharing is not applicable to this article as no datasets were generated during the current study.
